# Co-carcinogenesis: Human Papillomaviruses, Coal Tar Derivatives, and Squamous Cell Cervical Cancer

**DOI:** 10.3389/fmicb.2017.02253

**Published:** 2017-11-16

**Authors:** Harry W. Haverkos, Gregory P. Haverkos, Michael O’Mara

**Affiliations:** ^1^Department of Preventive Medicine and Biostatistics, Uniformed Services University of the Health Sciences, Bethesda, MD, United States; ^2^Pharmacist, Cincinnati, OH, United States; ^3^Chemist, Tarpon Springs, FL, United States

**Keywords:** co-carcinogenesis, human papillomavirus, cervical cancer, co-factors, cresols, cigarette smoke, coal tar

## Abstract

Cervical cancer (CC) is the fourth most common cancers among women worldwide. Human papillomaviruses (HPVs) play a major role in the etiology of CC, with several lines of epidemiologic and experimental evidence supporting a role for non-viral (co-carcinogens) and host genetic factors in controlling the risk for progression to neoplasia among HPV-infected individuals. The role of co-carcinogens in the development of CC is significant in the developing world where poor sanitation and other socio-economic conditions increase the infectious cancer burden. Here, we discuss how exposure to environmental factors such as coal tar derivatives from cigarette smoking, tar-based sanitary products, and inhaled smoke from biomass-burning stoves, could activate host pathways involved in development of HPV-associated squamous cell cancers in resource-limited settings. Understanding interactions between these pathways with certain oncogenic HPV genotypes may guide implementation of strategies for control and treatment of HPV-associated cancers that develop in populations at high risk of exposure to various co-carcinogens.

## Introduction

Cancer initiation and progression is driven by a series of changes in DNA that control gene expression, resulting in uncontrolled cellular proliferation. The mutation theory of cancer causation suggests that cancer-associated gene expression arises from random replication errors, exposures to carcinogens (e.g., viruses, radiation, cigarette smoke), or faulty DNA repair processes. Although HPV infection is necessary for cervical cancer (CC) development, progression to cancer occurs in only a small percentage of HPV-infected women, and a number of studies have shown that incident cervicovaginal HPV is self-limited disease generally lasting less than a year in duration (Ho et al., 1998; Woodman et al., 2001; Workowski et al., 2015). From our perspective, human papillomaviruses (HPVs) play a critical role in the etiology of CC, with several lines of epidemiologic and experimental evidence supporting a role for non-viral (co-carcinogens) in controlling the risk for progression to neoplasia among HPV-infected individuals (Bennett et al., 2010; Wei et al., 2014; Goodson et al., 2015). The studies cited here were identified from a systemic review we conducted (Haverkos et al., 2003), reviews conducted by others (Working Group on the Evaluation of Carcinogenic Risks to Humans [IARC], 2004; International Collaboration of Epidemiological Studies of Cervical Cancer [ICESCC] et al., 2006; International Collaboration of Epidemiological Studies of Cervical Cancer [ICESCC], 2007), and other papers that we have read.

## Human Papillomaviruses and Cervical Cancer

Papillomaviruses are double-stranded DNA viruses that infect skin and mucosa of vertebrates and induce cellular proliferation in a species-specific manner (Working Group on the Evaluation of Carcinogenic Risks to Humans [IARC], 2007). Over 200 genotypes of HPV, belonging to 49 species, in five genera have been described, and the rate of HPV genotype discovery is rapidly increasing (Working Group on the Evaluation of Carcinogenic Risks to Humans [IARC], 2007; Bernard et al., 2010; Bzhalava et al., 2015). Among them, HPV-16 and HPV-18 play a major role in the etiology of CC (Working Group on the Evaluation of Carcinogenic Risks to Humans [IARC], 1995, 2007; Walboomers et al., 1999; Bosch et al., 2002; International Collaboration of Epidemiological Studies of Cervical Cancer [ICESCC] et al., 2006; International Collaboration of Epidemiological Studies of Cervical Cancer [ICESCC], 2007). Over 95% of women with CC are infected with one or more HPV types, most commonly HPV-16 (present in about 50% of all CC) and HPV-18 (present in about 10–15% of cases). Approximately 20 other HPV types (i.e., HPV-45, HPV-31, HPV-59, HPV-58, HPV-33, and HPV-11) have also been associated with CC and thus deemed oncogenic (Working Group on the Evaluation of Carcinogenic Risks to Humans [IARC], 2007; Guardado-Estrada et al., 2014). Harald zur Hausen was awarded the 2008 Nobel Prize in Medicine for demonstrating a link between HPV infection and development of CC (zur Hausen, 1989).

While HPV is a major factor for developing CC, and given that persistent infection with a high-risk “oncogenic” type of HPV appears to be necessary for the development of invasive CC (Walboomers et al., 1999; Munoz et al., 2003), a number of studies have shown that squamous cell CC may result from an additive or synergistic interaction between oncogenic HPVs and cervical tar exposures, a process generally referred to as co-carcinogenesis (Haverkos, 2004; Working Group on the Evaluation of Carcinogenic Risks to Humans [IARC], 2004; International Collaboration of Epidemiological Studies of Cervical Cancer [ICESCC] et al., 2006; International Collaboration of Epidemiological Studies of Cervical Cancer [ICESCC], 2007). We highlight coal tar derivatives from cigarette smoking, tar-based vaginal sanitization products, and inhaled smoke from burning biomass (wood, coal, dung) in poorly ventilated kitchens, as potential co-carcinogenic factors that contain bioactive compounds likely to play a determinative role in CC development (Rotkin, 1967; Winkelstein, 1990; Ferrara et al., 2000; Haverkos et al., 2000, 2003; Velema et al., 2002; Steckley et al., 2003; Working Group on the Evaluation of Carcinogenic Risks to Humans [IARC], 2004; Haverkos, 2005; International Collaboration of Epidemiological Studies of Cervical Cancer [ICESCC] et al., 2006; International Collaboration of Epidemiological Studies of Cervical Cancer [ICESCC], 2007; Bennett et al., 2010). We identify published data from virological and genetic studies linking oncogenic papillomavirus genotypes and chemicals to development of cancer (Rous, 1965; Prokopczyk et al., 2009; Wei et al., 2014).

## Coal Tar Related Risk Factors for Cervical Cancer

### Cigarette Smoking

Using data from the Surveillance, Epidemiology, and End Results (SEER) study, Winkelstein colleagues proposed a potential role for cigarette smoke as a causative factor for CC, based on the results of two epidemiologic studies. First, he showed a direct correlation between the incidence rates of CC and male lung cancer in the United States (Winkelstein et al., 1977), and then reviewed four case-control studies, two from the United States and two from Great Britain, demonstrating that women who smoked cigarettes were more likely to develop CC (Winkelstein, 1977). Several groups have subsequently confirmed the links between cigarette smoking and CC (Haverkos et al., 2003; Steckley et al., 2003; International Collaboration of Epidemiological Studies of Cervical Cancer [ICESCC] et al., 2006); consequently, the International Agency for Research on Cancer (IARC) listed tobacco smoke as a cause of CC (Working Group on the Evaluation of Carcinogenic Risks to Humans [IARC], 2004). Furthermore, an international group of epidemiologists pooled data from 23 studies, representing over 13,000 women with CC and over 23,000 controls. Current smokers were at increases risk for squamous cell CC compared to women who never smoked (Relative rate = 1.60, 95% confidence intervals 1.48–1.73, *p* < 0.001). The risk was dose-dependent and increased for women who started smoking at earlier ages. Notably, the risk appeared to be specific for squamous cell CC and not for adenocarcinoma of the cervix (International Collaboration of Epidemiological Studies of Cervical Cancer [ICESCC] et al., 2006; International Collaboration of Epidemiological Studies of Cervical Cancer [ICESCC], 2007).

We conducted a meta-analytical and geographical variability evaluation by relevant variables in the United States and over 70 countries. Our results were similar to Winkelstein’s findings for United States and Europe; however, we found a negative correlation between smoking among women and CC (Pearson’s coefficient = -0.550). We proposed the need to search for other environmental factors as potential contributors to CC development among women in developing countries (Steckley et al., 2003).

### Cooking Practices

Three billion people worldwide depend on coal, crop residue, dung, and wood for cooking and heating. The highest proportion of populations using solid fuels include several African countries and other developing nations (World Health Organization [WHO], 2006), where the highest rates of CC have been reported. In fact, exposure to environmental smoke from burning biomass represents the most important source of bio-carcinogens worldwide that might explain the geographic variability of CC. Epidemiologists working in Honduras associated cooking over wood-burning stoves and CC (Ferrara et al., 2000; Velema et al., 2002). We pursued this link with another ecologic study and demonstrated that the Pearson coefficient for solid fuel use and CC was 0.498 (*p* < 0.05) (Bennett et al., 2010), further supporting a role for inhaling smoke from sources other than tobacco as a cofactor in CC development.

### Tar-Based Hygiene Products and CC: A Historical Perspective

Frank Smith, a NYC clinician, observed that women with CC used commercially available vaginal sanitization products (Lysol^®^) more frequently than other women in his practice. He described Lysol^®^ was a coal-tar derivative and noted that such tars were employed in animals to produce cancer (Smith, 1931; Waller, 1994; Fujiki, 2014). Epidemiologists in Massachusetts noted that vaginal cleaning with Lysol^®^ and other products containing carbolic acid, creolin, or sulfonaphthol, was reported more frequently by CC patients than others (Lombard and Potter, 1950). In California, Isadore Rotkin studied over 400 women with CC compared to hospital-based controls matched for age, race, and religion, and found a significant association vaginal sanitization products and cancer (Rotkin, 1967). By 1970 manufacturers of those products voluntarily discontinued distribution of those products in the United States, but interestingly, not worldwide (Kilmarx et al., 1998). We have proposed that those tar-based products placed directly on the cervix may produce similar changes as those due to cigarette smoking (Bennett et al., 2010).

## Genetic Alterations Associated With CC Development

Ames (1979) suggested that natural and man-made chemicals may contribute to the DNA damage that commits cells to a cancerous state. Carcinogen-induced mutations may also lead to chromosomal rearrangements including aneuploidy, a condition that arises during cell division when the chromosomes do not segregate properly between two daughter cells. An extra or missing chromosome is often seen in CC (McCormack et al., 2013).

Goodson et al. (2015) noted 85 examples of environmental chemicals that disrupted key pathways in carcinogenesis, designated as “hallmarks of cancer.” Those hallmarks include hyperproliferative signaling, insensitivity to growth-factor signals, evasion of apoptosis, sustained angiogenesis, genomic instability, and mutation, promotion of inflammation, and dysregulation of metabolism. Individual chemicals in mixtures, such as tobacco smoke and tar-based vaginal sanitary products, accumulate in cells and tissues and activate important carcinogenic pathways (Goodson et al., 2015).

Tobacco smoke is known to contain about 6,000 compounds, including benzyl pyrenes, polycyclic aromatic compounds, and tobacco specific nitrosamines. For example, benzo[a]pyrene have been identified at higher levels in cervical mucous of smokers than non-smokers (Melikian et al., 1999a). Several investigators have challenged HPV-infected cell lines with benzo[a]pyrene, a known carcinogenic component of cigarette smoke, and demonstrated increased viral titers and DNA damage measured by increased DNA adducts, E6 and E7 oncogene expression, and retinoblastoma protein (pRb) (Sizemore et al., 1995; Melikian et al., 1999b; Alam et al., 2008, 2010; Maher et al., 2011). Investigators in New Mexico exposed cervical cell lines containing episomal HPV genomes to tobacco smoke condensate and observed increased E6 and E7 oncogene expression, suggesting prominent roles for both HPV and tobacco smoke in cancer development (Wei et al., 2014) (**Table 1**).

**Table 1:** Co-carcinogenesis: *in vitro* studies of HPV-chemical interactions.

HPV	Chemical	Cell line	Primary result	Reference
16	B(a)P	HPV-immortalized epithelial cells	DNA damage, decreased p53 levels	Sizemore et al., 1995
16	B(a)P	Cervical cells	Increased DNA adducts and E6 expression	Melikian et al., 1999b
31	B(a)P	CIN tissue	Ten-fold increase in HPV titers	Alam et al., 2008
16	NNK	Human ectocervical cells	Alteration in expression of 30 different genes	Prokopczyk et al., 2009
31	B(a)P	CIN tissue	Increased pRb and cdc2/CDK1 activity	Alam et al., 2010
16	B(a)P	Caski cells	Increased E7 expression; curcumin may block effects	Maher et al., 2011
16, 31	MTSC	CIN tissue	Increased E6 and E7 oncogene expression	Wei et al., 2014

*B(a)P, benzo[a]pyrene; MTSC, mainstream tobacco smoke condensate; Caski cells, see Maher et al., 2011; CDK, cell cycle-specific cyclin-dependent kinase; CIN, cervical intraepithelial neoplasia biopsy; NNK, a tobacco specific nitrosamine, 4-(methylnitrosamino)-1-(3-pyridyl)-1-butanoone; pRb, retinoblastoma protein.*

## Other HPV-Related Cancers

Working Group on the Evaluation of Carcinogenic Risks to Humans [IARC] (2007) updated their evaluation of the carcinogenic risks of HPVs. They concluded that HPV-16 is carcinogenic for cancers of the anus, oral cavity, oropharynx, penis, vagina, and vulva (Working Group on the Evaluation of Carcinogenic Risks to Humans [IARC], 2007; Chaturvedi, 2010). However, whether tar-based factors could be involved in the pathogenesis of other HPV-related cancers remains to be determined. For example, investigators in Maryland conducted a case-control study of 100 patients (86 males, 14 females) with oropharyngeal squamous cell cancers and 200 age- and sex matched patients without cancer seen at the same outpatient otolaryngology clinic. Oropharyngeal cancer was strongly associated with oral HPV type 16 infection (Odds ratio, 14.6), and oral infection with any of 37 HPV types (OR, 12.3). HPV-16 DNA was found in 72 of 100 paraffin-embedded tumor specimens. Additionally, cancer was significantly associated with greater than 28 alcoholic drinks per week (OR, 4.1), more than 20 tobacco pack-years (OR, 2.4), and smoking marijuana monthly for more than 1 year (OR, 2.1). The authors conclude that oropharyngeal cancer is strongly associated with oral HPV infection with or without the established risk factors of alcohol or tobacco use (D’Souza et al., 2007). On the other hand, we propose that oral HPV infection and oral coal tar derivative exposures are component causes of oropharyngeal squamous cell cancers, similar to the co-carcinogenic process inducing CC.

## Discussion

Rous (1910) described the Rous sarcoma virus (RSV) as the causative agent for a sarcoma by inoculating a naïve chicken with a cell-free filtrate from another, infected chicken with sarcoma, and won the Nobel Prize in Medicine for it in 1966. However, some investigators questioned Rous’s attribution of the cancer to RSV alone, and insisted that the cancer was caused by the virus only after inflammation was induced by scraping the area with sand added to the filtrate (Gye, 1925, 1926, 1938; Gye and Mueller, 1929).

In an attempt to replicate Rous’s observations with sarcoma, William E. Gye conducted a series of experiments in chickens, and concluded:

“These researches have led me to look upon cancer – using the term in its widest sense – as a specific disease caused by a virus (or group of viruses). Under experimental conditions the virus alone is ineffective; a second specific factor, obtained from tumor extracts, ruptures the cell defenses and enables the virus to infect. Under natural condition continued “irritation” of tissues sets up a state under which infection can occur. The connection between the specific factor of a tumor and an irritant remains to be investigated. Some of the relatively unimportant “irritants” are known, such as coal-tar, paraffin oils, and etc. The virus probably lives and multiplies in the cell and provokes the cell to continued multiplication” (Gye, 1925).

These seminal studies set the stage for new thinking about the etiologic role of viruses and cancer development, but they also resulted in exciting new questions about whether a single oncogenic virus could be sufficient to cause cancer without the influence of other, contributing factors such as viral genetics, environmental irritants, and the immune status of the infected host.

The Rous-Gye debate led to Rous’s later works. Although squamous cell cancers had previously been produced by Kennaway using multiple doses of a polycyclic aromatic hydrocarbon (Waller, 1994; Fujiki, 2014), Rous and colleagues consistently demonstrated the joint action of tar and Shope papillomavirus (or cottontail rabbit PV, CRPV1), now referred to as Sylvilagus floridanus Papillomavirus 1 (SfPV1) (Bernard et al., 2010) in inducing squamous cell carcinomas in rabbits in shorter time periods. They painted rabbits’ ears or abdomens with methylcholanthrene once, before or after a single inoculation with a papillomavirus and noted cancer development within 3–6 months (Rous and Beard, 1934; Rous and Kidd, 1938; Rogers and Rous, 1951). Based on these observations, Rous proposed that cancer resulted from a two-step process of “initiation and promotion” (Rous, 1965).

While the Henle-Koch postulates for causation have been accepted as a basis for establishing that a virus may be causally linked to a specific cancer, Evans (1977, 1982) and Evans and Mueller (1990) recognized several problems that might slight the universal application of such a “standard.” Evans instead proposed that the cause(s) of cancers of any type more likely involve a complex interplay of infectious agents, chemicals, and other factors that modify susceptibility to the oncogenic state.

Working Group on the Evaluation of Carcinogenic Risks to Humans [IARC] (1995) an international body declared “at least” HPV-16 and HPV-18 cause CC. Later, scientists identified 17 other HPV types (26, 31, 33, 35, 39, 45, 51, 52, 53, 56, 58, 59, 66, 67, 68, 73, and 82) as “high-risk” for cancer (Munoz et al., 2003; de Villiers et al., 2004). In 2004, WHO recognized cigarette smoke as another causative agent for cervical cancer (Working Group on the Evaluation of Carcinogenic Risks to Humans [IARC], 2004). We added coal tar derivatives in vaginal douche products and by inhaling smoke from wood and coal burning stoves as probable co-carcinogens (Bennett et al., 2010).

We have formulated our virus-coal tar derivative hypothesis from observational studies. Epidemiologic investigations, especially ecologic studies, have well-known limitations. Case-control and cohort studies provide a higher level of evidence but are subject to confounding and various biases, i.e., recall and selection bias. Although epidemiologic studies may provide additional insights, such questionnaire-based research is not able to isolate individual carcinogens in tobacco and wood-burning smoke. For example, scientists have explored the interaction of benzo[a] pyrene and tobacco smoke condensate on HPV-immortalized cells. We encourage more studies combining “high risk” HPV types and coal tar derivatives, both individual chemicals and various mixtures, in the laboratory and observing effects on viral genetics and cancer induction. One might re-enact the Peyton Rous rabbit model with Shope papillomavirus and replacing methylcholanthrene with chronic administration of coal tar derivatives. Those studies should provide insights into the process of co-carcinogenesis involving viruses and chemicals. One might also find that this concept may apply to other cancers, including oropharyngeal and anal carcinomas.

An overwhelming body of evidence clearly identifies HPV as a major etiologic factor for CC, and we support universal immunization of boys and girls with HPV vaccines (Future II Study Group, 2007; Joura et al., 2015). In addition, Papinicoulaou testing with or without HPV-specific testing remains a life-saving intervention for many women (Schiffman and Solomon, 2013; Centers for Disease Control and Prevention [CDC], 2015). However, the etiology of CC appears to be complex and multi-factorial. Infection with HPV plays a central role in CC development, with host, viral, and environmental factors influencing the risk for progression to neoplasia. A growing number of reports on the pathogenesis of oncogenic HPV genotypes, the relative contributions of co-carcinogens, and the host genetic determinants of cancer development, provide a unifying perspective on etiology and pathogenesis of cancer. In addition, current studies demonstrate a viral genetic basis of pathogenicity in which oncogenic HPV types display unique fitness for persistence. Nonetheless, gaps remain in our understanding of the correlates of CC development, and future research is needed to delineate the genetic factors that influence cancer development. Such efforts should lead to even more effective strategies for prevention and treatment of CC in high risk populations.

## Author Contributions

HH developed concept and outline of the paper, wrote the first draft, provided infectious diseases expertise and cigarette smoking sections, and is the corresponding author. GH wrote historical section on pharmacologic aspects of vaginal douche products and reviewed all aspects of the paper. MO wrote chemical sections of coal tar and coal tar derivatives sections and reviewed all aspects of the paper.

## Disclaimer

The views expressed are those of the authors, and do not necessarily reflect the official views of the Uniformed Services University of the Health Sciences, or the Department of Defense (DOD).

## Conflict of Interest Statement

The authors declare that the research was conducted in the absence of any commercial or financial relationships that could be construed as a potential conflict of interest.
